# A novel tRNA variable number tandem repeat at human chromosome 1q23.3 is implicated as a boundary element based on conservation of a CTCF motif in mouse

**DOI:** 10.1093/nar/gku280

**Published:** 2014-04-21

**Authors:** Emily M. Darrow, Brian P. Chadwick

**Affiliations:** Department of Biological Science, Florida State University, Tallahassee, FL 32306-4295, USA

## Abstract

The human genome contains numerous large tandem repeats, many of which remain poorly characterized. Here we report a novel transfer RNA (tRNA) tandem repeat on human chromosome 1q23.3 that shows extensive copy number variation with 9–43 repeat units per allele and displays evidence of meiotic and mitotic instability. Each repeat unit consists of a 7.3 kb GC-rich sequence that binds the insulator protein CTCF and bears the chromatin hallmarks of a bivalent domain in human embryonic stem cells. A tRNA containing tandem repeat composed of at least three 7.6-kb GC-rich repeat units reside within a syntenic region of mouse chromosome 1. However, DNA sequence analysis reveals that, with the exception of the tRNA genes that account for less than 6% of a repeat unit, the remaining 7.2 kb is not conserved with the notable exception of a 24 base pair sequence corresponding to the CTCF binding site, suggesting an important role for this protein at the locus.

## INTRODUCTION

Almost two-thirds of the human genome is composed of repetitive DNA (1), a proportion of which corresponds to tandem repeats. Tandem repeats consist of DNA sequences organized into a head-to-tail arrangement, and size of the individual repeating unit varies from just a few base pairs (bp) in the case of microsatellites (2) to several kilobases (kb) for some of the largest tandem repeats in the human genome (3).

Only a handful of the large tandem repeats, or macrosatellites, are well characterized, with many corresponding to gaps in our genome sequence due to the inherent difficulty with the assembly of repeat DNA (4). Like most tandem repeats, the copy number of individual repeat units within a macrosatellite varies between individuals making macrosatellites some of the largest variable number tandem repeats (VNTRs) in the genome (3,5,6,7,8,9,10,11,12).

What role macrosatellites fulfill in our genome is unclear. Some contain open reading frames (ORFs) that are predominantly expressed in the testis or certain cancers (13,14,15), whereas the expression of others is more widespread (16,17,18,19). However, reduced copy number of some is associated with disease (5,20) due to inappropriate reactivation of expression (21). Others contain no obvious ORF (6,11); further complicating what purpose they serve. However, at least for the X-linked macrosatellite DXZ4 (6), a female-specific chromatin configuration adopted at the allele on the inactive X chromosome (22) mediates long-range chromosome interactions (23), suggesting that it might perform a structural role, contributing to the alternate 3D organization of the chromosome territory (24).

Here we report the characterization of a novel transfer RNA (tRNA) gene cluster on human chromosome 1q23.3 that consists of a large VNTR that is conserved in mammals and displays the hallmarks of a genomic boundary element.

## MATERIALS AND METHODS

### Cell lines

All CEPH lymphoblastoid cell lines (LCLs) were obtained from the Coriell Institute for Medical Research (www.coriell.org), as were LCLs used in the copy number variation (CNV) panels. Primate primary fibroblast cells: Rhesus Macaque (AG08305 and AG08312), Pig-Tailed Macaque (AG07921 and AG08312), Common Squirrel Monkey (AG05311) and the Black-Handed Spider Monkey (AG05352) were obtained from Coriell. The Gorilla LCLs were a gift from H. Willard (Duke University). Human fibroblast and epithelial cell lines were obtained from the American Type Culture Collection (www.atcc.org). Cells were maintained according to the recommendations of the suppliers.

### Plug preparation

In preparation, a molten stock of 1.0% (w/v) low-melting temperature agarose was prepared in L-buffer (100 mM EDTA pH8.0, 10 mM Tris-Cl pH7.6, 20 mM NaCl) and kept at 42°C. Single-cell suspensions of LCLs were prepared by pipetting cultures up and down, whereas fibroblast and epithelial suspensions were prepared by removing monolayers of cells from culture vessels by trypsin treatment. Cells were suspended at 2 × 10^7^ cells/ml in L-buffer, and equilibrated to 42°C for 5 min. The cells were briefly resuspended and mixed 1:1 with 42°C low-melting temperature agarose and applied to the plug mold (∼80 μl per slot). The mold was placed at 4°C for at least 30 min to solidify before transfer of the plugs to 3 volumes of L-buffer supplemented with 1 mg/ml proteinase K and 1% sarkosyl. Plugs were incubated at 50°C for 3 h, before replacing with fresh digestion mix and returning to 50°C overnight. The following day, plugs were cooled to room temperature and rinsed twice with ultrapure water followed by three 60-min washes in 50 volumes of TE buffer (10 mM Tris-Cl pH8.0, 1 mM EDTA pH8.0). Plugs were stored at 4°C in 10 volumes of TE buffer (10mM Tris-HCl, 1mM EDTA, pH8.0).

### Pulsed field gel electrophoresis

A single plug for each sample was transferred to a 1.5 ml tube and equilibrated for 20 min at room temperature in 1× New England Biolabs (NEB) buffer-2 supplemented with 1× bovine serum albumen (BSA) buffer (New England Biolabs, MA, USA). The buffer was removed and replaced with 200 μl of 1× NEB buffer-2/1× BSA containing 400 units of XbaI restriction endonuclease (New England Biolabs, Ipswich, MA, USA). Digests were performed overnight at 37°C. A 1.0% agarose gel was prepared in 0.5× Tris-borate-EDTA (TBE) buffer (50 mM Tris-Cl pH8.3, 50 mM boric acid, 1 mM EDTA) using PFGE-certified agarose (Bio-Rad Laboratories, Hercules, CA, USA). Samples were separated in 0.5× TBE at 14°C using a Bio-Rad CHEF Mapper XA System (Bio-Rad Laboratories, Hercules, CA, USA) set to resolve DNA fragments of 100–400 kb. Upon run completion, the gel was transferred to a solution of 1 μg/ml ethidium bromide in ultrapure water for 30 min at room temperature, before destaining with two 15-min washes with ultrapure water. Images were captured and the migration distance of the molecular weight markers was measured in millimeters. Molecular weight markers include MidRange PFG Marker I and II (New England Biolabs, MA, USA), and Lambda HindIII marker (Life Technologies, Grand Island, NY, USA).

### Southern blotting and hybridization

The gel was immersed for 15 min at room temperature in 0.25M HCl, before rinsing and soaking in denaturing solution (1.5 M NaCl, 0.5 M NaOH) for 30 min at room temperature. Southern blotting was performed essentially as described (25), transferring DNA overnight to Hybond N+ (GE Healthcare Bio-Sciences, Pittsburgh, PA, USA). The orientation of the blot and well location were marked with a soft pencil before rinsing in 2× saline-sodium citrate buffer (SSC) (300 mM NaCl, 30 mM sodium citrate, pH7.0) followed by baking at 120°C for 30 min.

Hybridization was performed at 60°C overnight in ExpressHyb (Clontech Laboratories Inc., Mountain View, CA, USA). The blot was prehybridized for 30 min. The probe (25 ng/ml of hybridization buffer) was denatured at 95°C for 8 min, quenched on ice for 2 min before adding to the prehybridization mix and incubating overnight in a rotating oven. The blot was washed at 60°C for 8 min twice with wash one (2× SSC, 0.1% sodium dodecyl sulphate (SDS)) and twice with wash two (0.2× SSC, 0.1% SDS). Digoxigenin-dUTP probes were detected using the DIG High Prime DNA Labeling and Detection Starter Kit II according to the manufacturers instructions (Roche Applied Science, Indianapolis, IN, USA).

### Probe preparation

A probe for Southern hybridization was prepared by polymerase chain reaction (PCR) amplification of a 519 bp fragment of the array using the following primers: Forward–CCGCGACCCTCTACCAATTG, Reverse–TGCTCAGCGGTCAGAAGTTG (Eurofins MWG Operon, Huntsville, AL, USA). PCR was performed on 100 ng of genomic DNA template using HotStar Taq (Qiagen, Germantown, MD, USA) with the following cycle: 10 min at 95°C, followed by 40 cycles of 95°C for 20 s, 58°C for 20 s and 72°C for 30 s. The PCR product was cleaned using the Qiaquick PCR purification kit (Qiagen, Germantown, MD, USA). Digoxigenin-dUTP probes were prepared using the DIG High Prime DNA Labeling and Detection Starter Kit II according to the manufacturers instructions (Roche Applied Science, Indianapolis, IN, USA).

## RESULTS

### A tRNA gene cluster at 1q23.3 is organized into a large GC-rich tandem repeat that is flanked by ERV LTR elements

**Figure 1.**
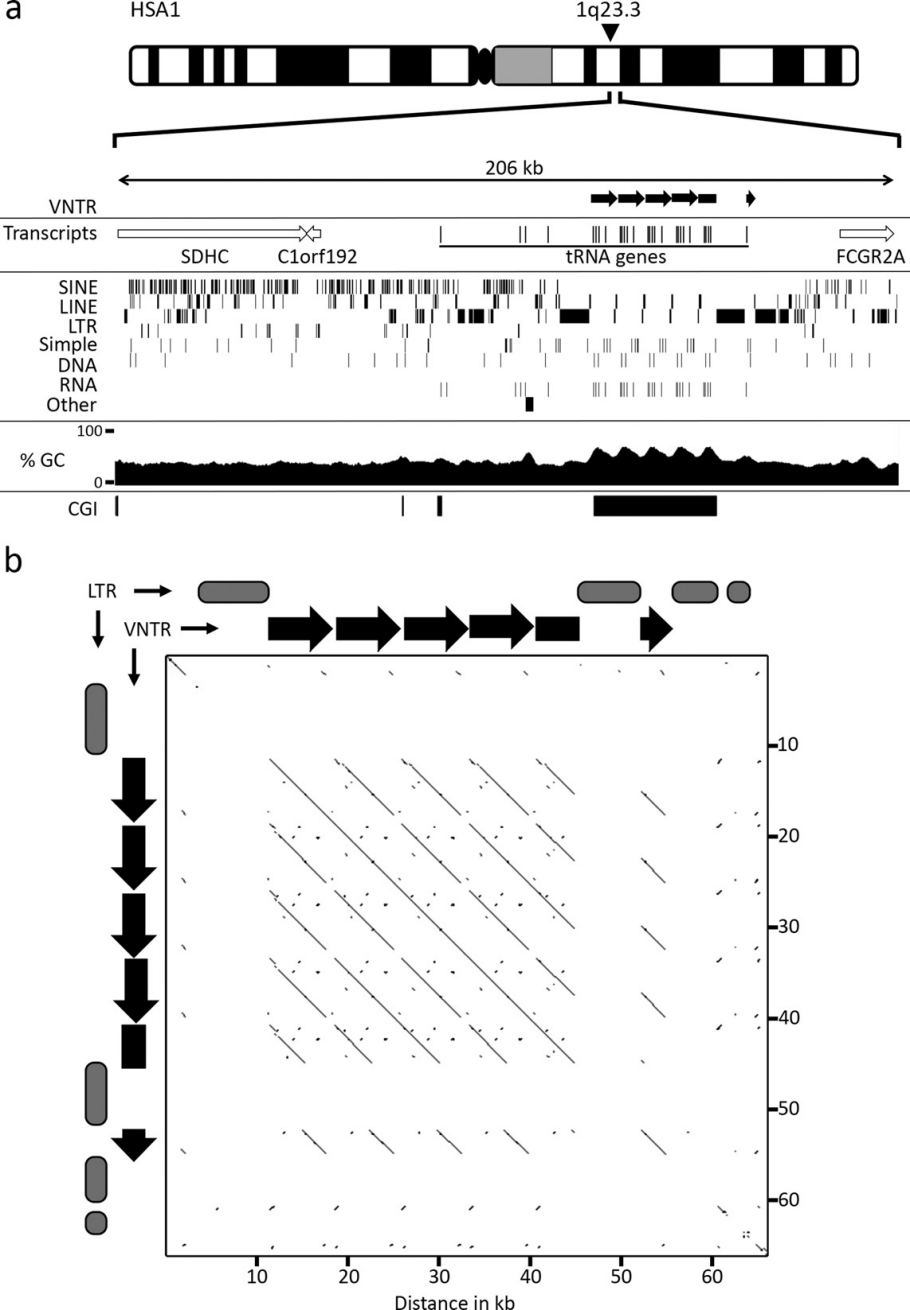
Genomic location and tandem arrangement of the tRNA cluster. (a) Ideogram of human chromosome 1, indicating the approximate location of the tRNA tandem repeat at 1q23.3. Immediately below is schematic map showing a 206 kb genomic window in the vicinity of the tRNA cluster. The location of the VNTR is indicated in the top section by the black right-facing arrows. Transcripts from the interval are indicated in the second section. Open arrows indicate the genomic coverage of the transcript and direction of transcription. Beneath this is a map showing the location of the indicated repeat types (left side labels). The next section shows a plot of GC percentage across the interval. The final section shows the location of CGI indicated by solid black boxes. (b) Pair-wise alignment of the VNTR and flanking LTR elements using YASS (www.http://bioinfo.lifl.fr/yass/index.php). The locations of the individual VNTR repeat units are represented above and to the left of the plot by black arrows. Gray oval blocks represent the location of LTRs that are excluded from the plot, indicated by gaps in the diagonal line.

We examined the human genome (GRCh37/hg19) using the University of California Santa Cruz (UCSC) Genome Browser (26) for the presence of large tandem repeats that displayed high GC content and a signature of repeat units arranged in tandem based on Repeat Masker output. A 33-kb region of human chromosome 1q23.3 satisfied both of these criteria, with a clear tandem repeat signature and a GC content of 64.2% (Figure 1a), which is substantially higher than the 41.0% average for chromosome 1 (27) and is annotated as an extensive CpG island (CGI) (28). Pair-wise alignment of the DNA sequence confirms that this is indeed a well conserved tandem repeat (Figure 1b), with individual repeating units sharing 98% DNA sequence identity. Notably, immediately flanking the tandem array are long terminal repeat (LTR) elements of endogenous retroviruses (ERV). The proximal edge is characterized by an ERVL element, whereas the distal edge contains LTRs of members of the ERV1 and ERVK families. In addition, a small fragment of an ERVK member is present in each repeat unit. LTR members of the ERV family constitute a small fraction of chromosome 1 (1.40% for ERVL, 2.80% for ERV1 and 0.31% for ERVK) (27), making this region enriched for LTR elements (38.0%).

Many large tandem repeats are expressed (11,12,22), with some coding for proteins (5,11,29). Examination of transcripts originating from the tandem repeat (30) revealed that several tRNA genes are embedded in each repeat unit as well as in the proximal genomic interval (Figure 1a).

**Figure 2.**
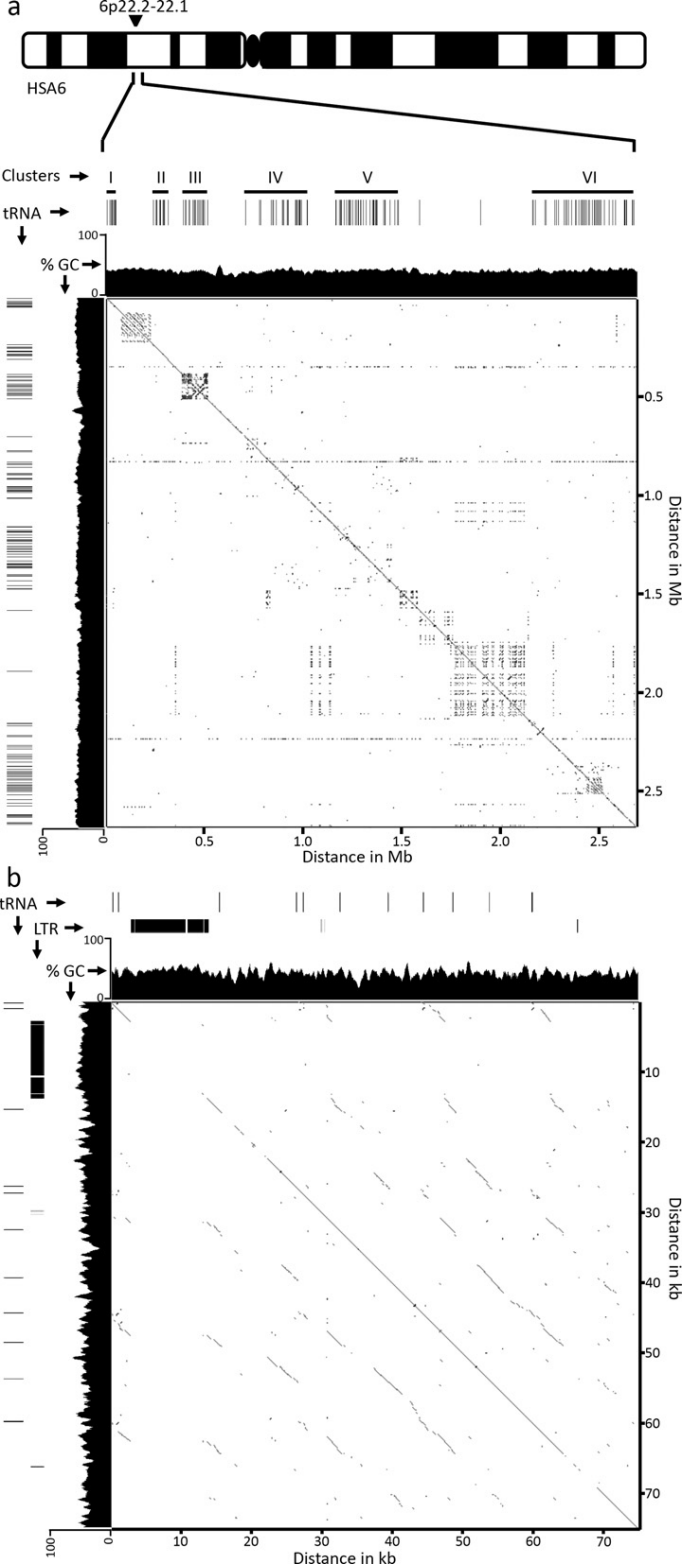
Genomic location of the major tRNA clusters on chromosome 6. (a) Ideogram of human chromosome 6, indicating the approximate location of the 6 tRNA clusters (I–VI) spread through a 2.7-Mb region corresponding to 26,284,348–28,973,050 of build GRCh37/hg19. Solid black lines within the clusters represent tRNA genes. A plot of GC percentage is shown immediately beneath the tRNA genes. Self-homology from the region is shown as a pair-wise alignment using YASS (www.http://bioinfo.lifl.fr/yass/index.php). (b) Pair-wise alignment of the cluster VI subregion that contains a sequence arranged in tandem, corresponding to 28,731,201–28,806,200 of build GRCh37/hg19. The location of tRNA genes and LTR elements within this 75-kb region are indicated, as is a plot showing the percentage of GC content.

An extensive tRNA gene cluster (tDNA) has been reported at human chromosome 6p22.2–22.1 (31), which contains six main clusters of tRNA genes that we have annotated as clusters I–VI (Figure 2a). Therefore, we examined this interval to see if high LTR and GC content were common for tRNA gene clusters. DNA sequence analysis indicates that the interval has a GC content of 41.1%, which is in line with the genome average of 41.0% (32). Furthermore, the GC distribution does not dramatically vary across the region and remains around 41% for each of the tDNA clusters (Figure 2a). Although not as high as observed at the chromosome 1 cluster, the overall LTR content of the 2.7 Mb interval is higher than the chromosome 6 average (13.28% compared to 8.05%) (31), and may reflect a relationship between these two genomic features.

Next, we examined the 2.7-Mb interval for evidence of tandem repeat arrangement, and identified several large sequences arranged tandem. However, when compared to the distribution of tRNA genes in the interval, neither of the two largest tandem repeats correlated with tDNA and instead corresponded to the Butyrophilin subfamily 2 immunoglobulin superfamily gene cluster (located between clusters I and II), and the Zinc finger SCAN domain containing gene cluster (located between clusters V and VI). A limited pattern of tandem and inverted DNA arrangement can be found at cluster III (Figure 2a), but displays broken homology and is largely due to a single duplication event with high long interspersed element (LINE) content (35.19% compared to 20.85% average for chromosome 6). A second region showing signatures of tandem arrangement is contained within cluster VI. This 75-kb region is 43.4% GC, but does show reasonable tandem arrangement and the presence of an 11-kb ERV1 element accounting for 14.84% of the interval. Excluding the cluster VI subregion, the highly conserved tandem arrangement, high GC sequence content and flanking LTR elements are unique to the tDNA cluster at chromosome 1q23.3.

### Characterization of the chromosome 1 tDNA tandem repeat unit

**Figure 3.**
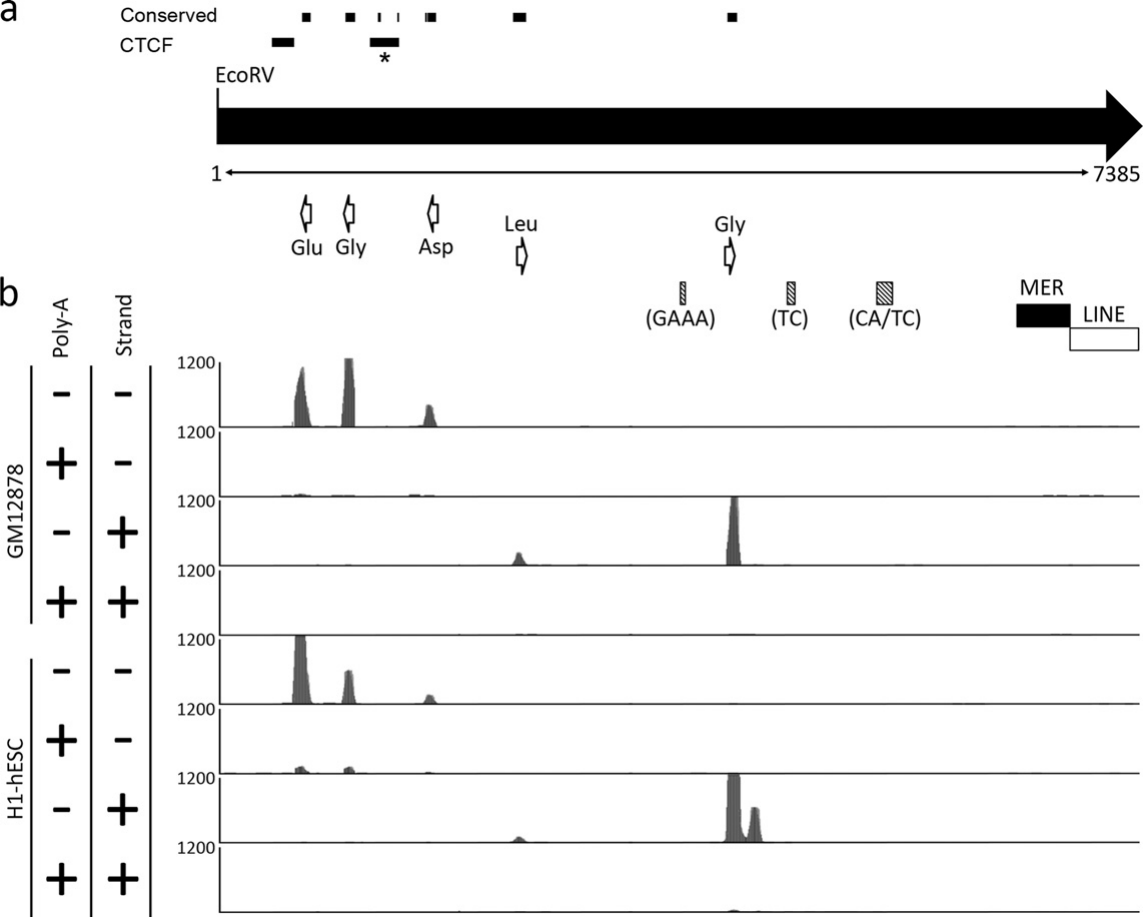
Characterization of a single repeat unit from the human chromosome 1q23.3 tRNA tandem repeat: genomic features and transcriptional units. (a) Schematic map of a single repeat unit, represented by the right-facing black arrow as defined by the periodicity of the restriction endonuclease *Eco*RV. The size of the repeat is indicated in bp immediately below the black arrow. The location and direction of transcription of tRNA genes are indicated by the open white arrows. The location of microsatellite repeats are indicated by the shaded gray boxes and the sequence composition of the repeat units indicated below each in brackets. The black and white boxes indicate the location of a MER and LINE element, respectively. Regions of conserved DNA sequence with the mouse repeat are indicated above the monomer, as are the location of two CTCF peaks. The peak marked with the * corresponds to the CTCF motif that is shared with mouse. (b) Representation of transcripts originating from a single repeat unit using data obtained from the ENCODE project (34). Image is adapted from the UCSC Genome Browser (www.genome.ucsc.edu) (26); build GRCh37/hg19 showing the track for Long RNA-seq from ENCODE/Cold Spring Harbor Lab. Data from the LCL GM12878 and hESC cell line H1 are indicated to the left. Polyadenylated RNA and nonpolyadenylated RNA are indicated by the ‘+’ and ‘−’ symbols, as are transcripts originating from the sense (+) and antisense (−) strands. Each track shows a vertical viewing range of 1200 reads.

Repeat units within the chromosome 1q23.3 tDNA tandem repeat share 98% DNA sequence identity. Variation between adjacent repeat units is due to single nucleotide polymorphisms within the unique sequence (77.8% of a single 7.2-kb monomer is unique and not repeat masked) or due to polymorphisms in the copy number of one of four simple tandem repeats composed of (GAAA), (TC), (CA) and (TC). Each repeat unit also contains a MER LTR fragment (5.7%) and a partial LINE element (7.37%), which are illustrated in Figure 3a. Each repeat unit contains five tRNA genes: a tRNA^Leu^ and tRNA^Gly^ encoded on the sense strand, and a tRNA^Glu^, tRNA^Gly^ and tRNA^Asp^ encoded on the antisense strand.

Transfer RNA genes are transcribed by RNA polymerase III (Pol III), and the transcripts are not polyadenylated (33). Examination of RNA-seq data from ENCODE (34) clearly shows that short, poly-A minus transcripts align precisely with the location of the tRNA genes and originate from the anticipated strand based on tRNA gene orientation (Figure 3b). Little to no transcript is detected in the poly-A plus fraction and no other transcriptional units are detected within the repeat unit, indicating that tRNAs are the only transcripts originating from the tandem repeat units. However, it is important to point out that there are multiple copies of the different tRNA genes scattered throughout the genome, many of which are identical in sequence. Therefore, RNA-seq reads assigned to the tRNA tandem repeat could conceivably have originated from an identical tRNA gene located on another chromosome. For example, the tRNA^Glu^ located in the array is 100% identical to a tRNA^Glu^ on chromosome 6. Nevertheless, the DNA sequence of the tRNA^Gly^ located adjacent to the tRNA^Glu^ is sufficiently different from other tRNA^Gly^ genes that its gene sequence is unique to the tRNA tandem array. Therefore, the aligned RNA-seq reads for at least this gene likely originate from the array.

### The tDNA cluster is bound by CTCF and characterized by both euchromatin and heterochromatin markers

**Figure 4.**
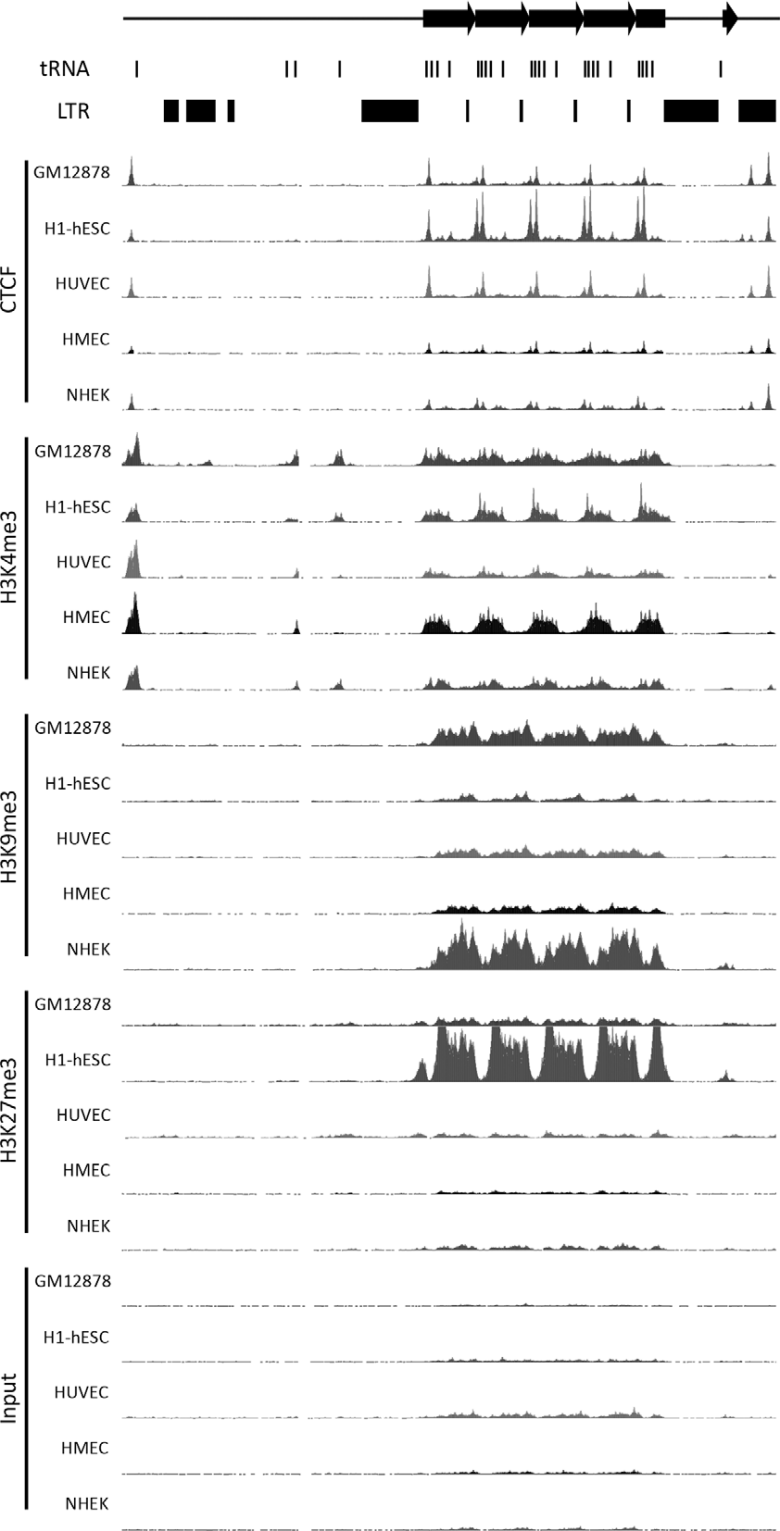
Chromatin organization at and around the tDNA tandem repeat. Image represents a 90-kb window covering 161,368,001–161,458,000 of human chromosome 1 generated from build GRCh37/hg19. Image adapted from the UCSC genome browser (26). The location of the VNTR is indicated at top by the right-facing black arrows. The location of tRNA genes and LTR elements is indicated immediately below this. Data from chromatin immunoprecipitation coupled with massively paralleled DNA sequencing is indicated to the left for CTCF, H3K4me3, H3K9me3, H3K27me3 and the input control (44). Data are shown for LCL GM12878, hESC cell line H1, human umbilical vein endothelial cells (HUVEC), human mammary epithelial cells (HMEC) and normal human epidermal keratinocytes (NHEK) as indicated to the left of each track. Each track has maximum vertical viewing range of 250 reads.

Most large tandem repeats are arranged into heterochromatin (22,35,36,37). Notable exceptions include the X-linked macrosatellite DXZ4 (6), that adopts both euchromatin and heterochromatin arrangements in response to X chromosome inactivation (22,23), and the chromosome 4 macrosatellite D4Z4, that adopts a more euchromatic organization in response to a reduction in the tandem repeat copy number (37,38), or due to haploinsufficiency of the heterochromatin protein SMCHD1 (39). Derepression of D4Z4 is directly correlated with onset of the progressive muscle wasting disease facioscapulohumeral muscular dystrophy (40). Similar to the chromosome 1 tDNA tandem repeat, both DXZ4 and D4Z4 are GC rich with 62.2% and 72.6% GC content, respectively (41). In addition, the multifunctional epigenetic organizer protein CCCTC-binding factor (CTCF) (42) can associate with both DXZ4 and D4Z4 (22,43). Given the similarities between the tDNA tandem repeat and the macrosatellites DXZ4 and D4Z4, we used publically available ENCODE data (44) to examine chromatin features of the tDNA cluster. Intriguingly, CTCF associates with two sites within each repeat monomer in all cell types examined, a selection of which are shown in Figure 4. Additionally, this tandem repeat is characterized by both histone H3 trimethylated at lysine 4 (H3K4me3) and histone H3 trimethylated at lysine 9 (H3K9me3), extending the parallels between the tDNA tandem repeat and the macrosatellite DXZ4 and D4Z4. H3K4me3 is a chromatin modification associated with active transcription (45), whereas H3K9me3 is associated with heterochromatin (46,47). An additional repressive chromatin modification is histone H3 trimethylated at lysine 27 (H3K27me3) that is catalyzed by the histone methyltransferase Enhancer of Zeste 2 (48,49,50,51). Surprisingly, high levels of H3K27me3 are associated with the tDNA tandem repeat, but only in human embryonic stem cells (hESC) (Figure 4). The hESC-specific association of both H3K4me3 and H3K27me3 with the tDNA repeat indicates that it is likely a bivalent domain (52) and suggests it may have an important role in development. A hESC-specific H3K27me3 signature also marks the chromosome 6 subregion of cluster VI that is arranged into a tandem repeat, but none of the other chromosome 6 tDNA clusters (data not shown). Although H3K4me3 is also a feature of this same region of cluster VI, the signature is much less extensive than at chromosome 1 and is centered on the individual tRNA genes and not throughout the tandem arranged DNA (data not shown).

### The chromosome 1 tDNA tandem repeat shows extensive CNV

**Figure 5.**
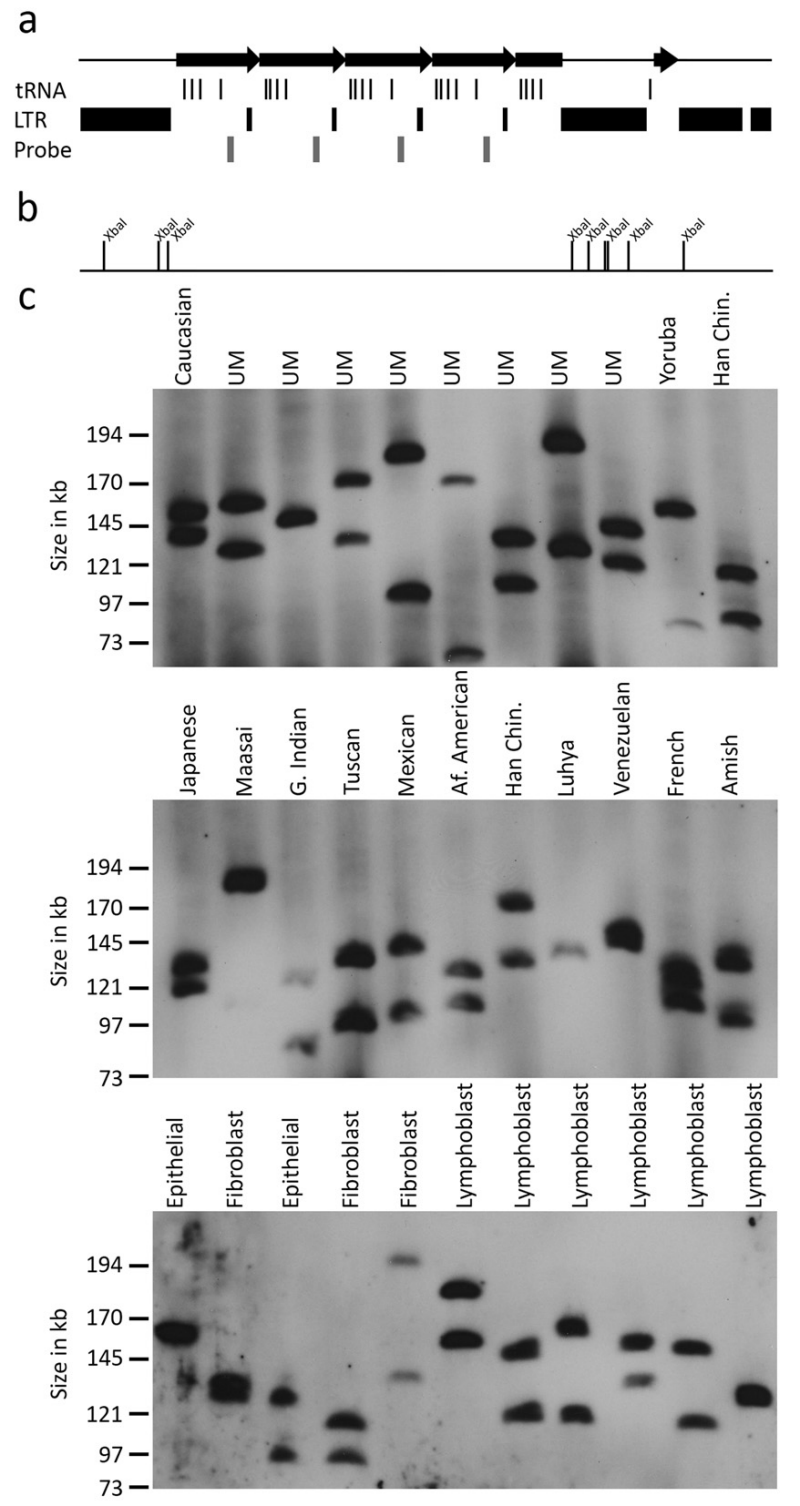
CNV of the tRNA tandem repeat. (a) Schematic map covering genomic coordinates 161,400,901–161,460,900 of chromosome 1 from build GRCh37/hg19. The black right-facing arrows indicate the location of the tRNA repeat. The location of tRNA genes and LTR elements are indicated beneath this, followed by the location of the 519 bp probe. (b) Restriction map of the same interval showing the location of all XbaI recognition sites. (c) CNV of the tRNA repeat. Images show Southern blot hybridization of XbaI cut DNA from 33 unrelated individuals, separated by PFGE and hybridized with the 519 bp probe. The ethnicity of samples is indicated above each lane (UM, Utah Mormon; Han Chin, Han Chinese; Af. American, African American; G. Indian, Gujarat Indian). Samples of unknown ethnic origins are labeled according to their corresponding cell type. The migratory size of molecular weight marker fragments is indicated on the left.

Most large tandem repeats in the human genome are polymorphic and therefore VNTRs (3,6,7,9,10,11,12). Some noncoding RNA genes are also arranged into large polymorphic tandem repeats, such as ribosomal DNA (53). Here, we have reported the first tandem repeat arrangement for some of the tRNA genes in humans, however, whether the tandem repeats display CNV is not known. Over 500 tRNA genes are located throughout the human genome, and analysis of sequencing data from the 1000-genome project (54) revealed that these genes are subject to evolutionary change as new tRNA genes were identified in some individuals and not others. Furthermore, analysis of relative number of sequencing reads from genome-wide sequencing data sets suggested that the copy number of some tRNA genes was higher than that annotated in the latest human genome build, including those located at 1q23.3 (55). In order to assess CNV at the 1q23.3 tDNA tandem repeat, we hybridized Southern blots of XbaI cut DNA that was separated by pulsed field gel electrophoresis (PFGE) with a probe contained within each repeat unit (Figure 5a). The location of the probe was selected to avoid any tRNA genes and to be unique to chromosome 1, and was first tested on an EcoRI genomic Southern blot to ensure that the anticipated single monomer fragment signal was detected and no other cross-hybridizing sequences (Supplementary Figure S1). XbaI was selected for the PFGE digest because there are no XbaI recognition sites within the tandem repeat, but XbaI sites are found immediately flanking the tandem array (Figure 5b). If the tandem repeat is not polymorphic, we would expect to see a 35-kb band in all individuals based on the most current build of the human genome. However, in 33 unrelated individuals of diverse ethnic background, we saw extensive CNV, with alleles ranging from 70 to 200 kb, indicating tandem repeat alleles composed of between 9 and 27 repeat units (Figure 5c).

### Altered VNTR allele size indicates meiotic and mitotic instability of the tDNA tandem repeat

**Figure 6.**
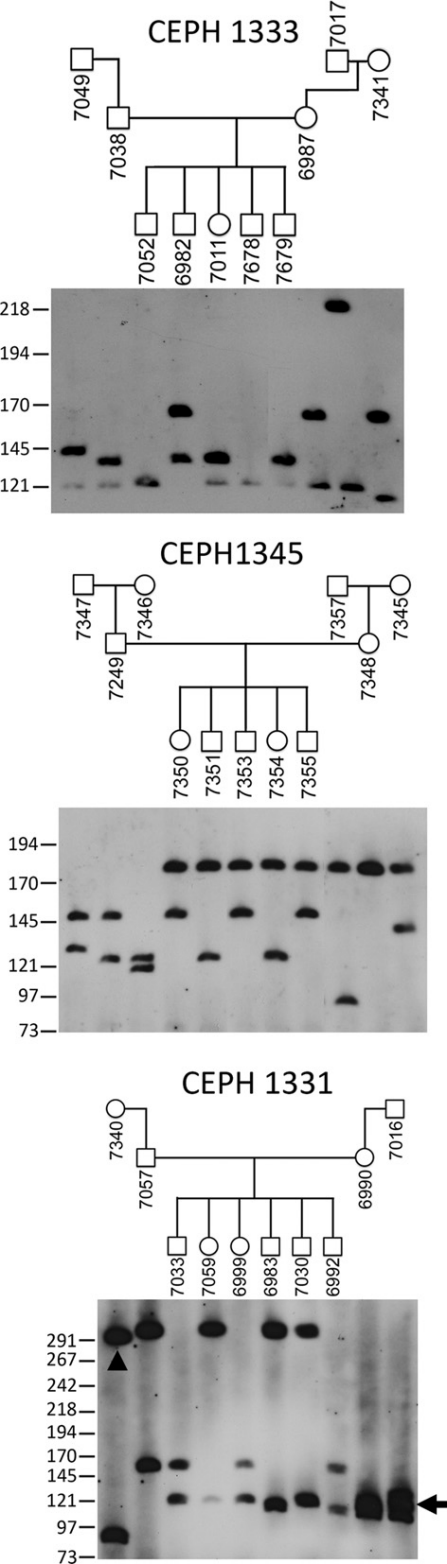
Mendelian inheritance and unstable transmission of the tRNA VNTR. Inheritance of the tRNA VNTR is shown for three CEPH Utah pedigrees: CEPH1333 (top), CEPH1345 (middle) and CEPH1331 (bottom). The identity and relationship of family members is indicated above each blot in the pedigrees. Member names are the Coriell GM0-ID for each individual. Alleles showing altered size are indicated by the arrowhead (meiotic instability) and arrow (mitotic instability). The migratory size of molecular weight marker fragments is indicated on the left.

Given the polymorphic nature of the tDNA VNTR, we sought to determine if alleles showed stable Mendelian inheritance and retention of allele size in culture. In order to do this, we determined allele sizes for the VNTR in three independent CEPH families across three generations. In two families (CEPH 1333 and CEPH1345) no allele size change was observed within individuals or between generations (Figure 6, top two panels). However, in family CEPH1331 evidence of meiotic instability was observed in grandmother 7340 as indicated by the increased allele size inherited by son 7057. In addition, grandfather 7016 shows evidence of mitotic instability by the presence of three alleles (Figure 6, bottom panel). Notably, CEPH1331 family also shows the largest allele size for the tDNA VNTR at 310 kb, which translates to ∼43 copies of the 7.3 kb tandem repeat unit.

### The tDNA tandem repeat is conserved in primates and demonstrates CNV

**Figure 7.**
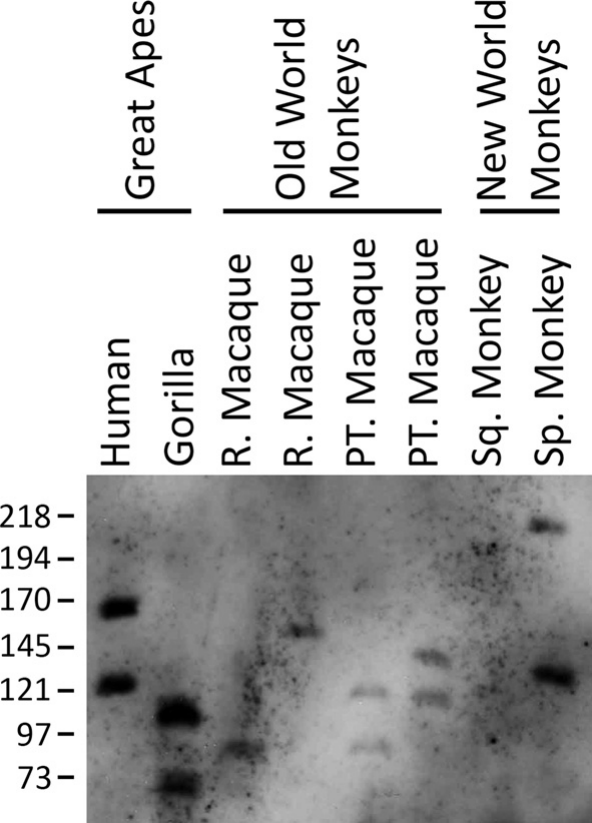
CNV of the tRNA VNTR in primates. Image shows a Southern blot of XbaI digested genomic DNA, separated by PFGE, hybridized with the 519bp probe. Samples include a human control, Rhesus Macaque (R. Macaque), Pig-Tailed Macaque (PT. Macaque), Common Squirrel Monkey (Sq. Monkey) and Black-Handed Spider Monkey (Sp. Monkey). Grouping of primate genealogy is indicated above. The migratory size of molecular weight marker fragments is indicated on the left.

The macrosatellite DXZ4 diverges rapidly through the primate lineage, but is sufficiently conserved to where it can be detected by hybridization, and confirmed to be a polymorphic VNTR in the Old World and New World monkeys (56). Therefore, the same probe that revealed CNV of the chromosome 1q23.3 tDNA tandem repeat in humans was used to assess a Southern blot of XbaI cut pulsed field gel separated DNA of primate samples from the Great Apes, Old World monkeys and New World monkeys. The probe target shares 95.6% sequence identity over 519 bp to Gorilla (comparison to build gorGor3), 87.4% sequence identity over 501 bp to Macaque (comparison to build rheMac3) and 84.4% sequence identity over 424 bp to Squirrel Monkey (comparison to build saiBol1), which likely reflects the weaker signals in the Old and New World monkey samples (Figure 7). Nevertheless, the large number of variable-sized alleles detected within and between primates indicates that the tDNA tandem repeat is a VNTR.

### The genomic and gene organization is conserved in mouse, but DNA sequence conservation is restricted to the tRNA genes and a CTCF binding site

**Figure 8.**
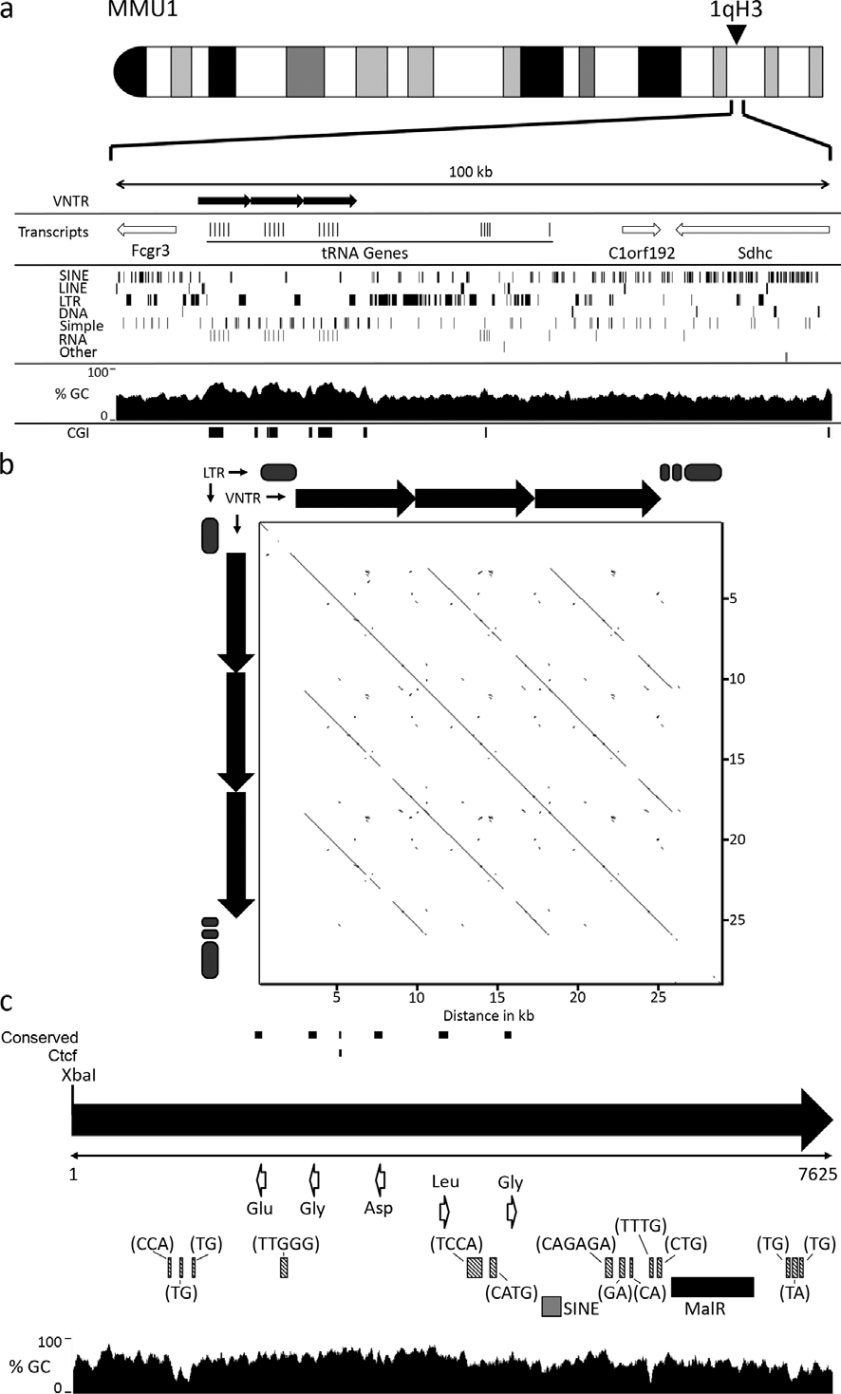
Characterization of the mouse tRNA cluster. (a) Ideogram of mouse chromosome 1, indicating the approximate location of the tRNA tandem repeat at 1qH3. Immediately below is schematic map showing a 100-kb genomic window in the vicinity of the tRNA cluster (corresponding to 172,981,160–173,081,065 of mouse chromosome 1, GRCm38/mm10). The location of the VNTR is indicated in the top section by the black right-facing arrows. Transcripts from the interval are indicated in the second section. Open arrows indicate the genomic coverage of the transcript and direction of transcription. Beneath this is a map showing the location of the indicated repeat types (left side labels). The next section shows a plot of GC percentage across the interval. The final section shows the location of CGI indicated by solid black boxes. (b) Pair-wise alignment of the VNTR and flanking LTR elements using YASS (www.http://bioinfo.lifl.fr/yass/index.php) corresponding to 172,990,701–173,020,200 of mouse chromosome 1 (GRCm38/mm10). The location of individual VNTR repeat units are represented above and to the left of the plot by black arrows. Gray oval blocks represent the location of LTRs that are excluded from the plot, indicated by gaps in the diagonal line. (c) Schematic map of a single repeat unit, represented by the right-facing black arrow as defined by the periodicity of the restriction endonuclease XbaI, corresponding to 173,000,076–173,007,700 of mouse chromosome 1 (GRC38/mm10). The size of the repeat is indicated in bp immediately below the black arrow. DNA sequences that are conserved with the human repeat are indicated as black lines above the black arrow, as is the conserved Ctcf binding motif. The location and direction of transcription of tRNA genes are indicated by the open white arrows. The location of microsatellite repeats are indicated by the shaded gray boxes and the sequence composition of the repeat units indicated in brackets. The black and gray boxes indicate the location of a MalR and SINE element, respectively.

Homologues of the DXZ4 and D4Z4 macrosatellites have been described in mouse (57,58). At least for DXZ4, DNA sequence conservation in mouse is restricted to the CTCF binding site. However, despite a lack of sequence conservation outside of this motif, the mouse Dxz4 locus is composed of a large tandem repeat with high GC content that is located in a syntenic region of the mouse X chromosome (58). Therefore, we sought to determine if the tDNA VNTR exists in the mouse genome, and if it does, what features of the human tandem repeat are conserved in mouse? The 7380-bp DNA sequence of a single human repeat unit was compared to the most recent build of the mouse genome (GRCm38/mm10). The first five matches all corresponded to a 70-kb interval of mouse chromosome 1qH3. This region is syntenic to human chromosome 1q23.3 (59) and the location of the human tDNA cluster (Figure 1a), with the matches residing between the *Fcgr3* and *C1orf192* and *Sdhc* genes (Figure 8a). Intriguingly, close examination of the DNA matches between the human 7380 bp sequence and the corresponding mouse DNA revealed that sequence identity was restricted to six short DNA sequences. Five of these DNA sequences correspond exactly to the same five tRNA genes as found at the human tDNA tandem repeat, whereas the sixth hit was 92% identical (22 of 24 nucleotides) to one of the two human CTCF peaks shown in Figure 4. The mouse and human sequences (Mouse: CGAGAGCGCCCCAGAGGAAAGGCG; Human: CGAGAGCGCCCCCAGAGGCAGGCG) each are a perfect match with the CTCF-binding motif determined by ChIP-seq (60) (Supplementary Figure S2). Consistent with these data, a single peak of Ctcf occupancy resides in the vicinity of this motif at the mouse tDNA repeat (Supplementary Figure S3). In contrast to mouse, the human repeat unit displays two distinct peaks of CTCF occupancy (Figure 4), only one of which overlaps a shared DNA sequence that matches a CTCF motif. It is possible that the second CTCF site in humans has arisen through a combination of the differential repeat element content of the human repeat unit compared to mouse along with the expansion of the repeat copy number, as this appears to be a common mechanism for the introduction of lineage-specific CTCF binding sites (61). Similar to the human tandem repeat (Figure 4), a peak of H3K4me3 defines the same region of the repeat, but at least in mouse, the H3K4me3 signal does not spread across the monomer. In contrast to the human locus (Figure 4), H3K27me3 enrichment is not obvious in mouse embryonic stem cells. Furthermore, H3K9me3 is not a prominent feature of the mouse repeat (Supplementary Figure S3), mirroring observations made between DXZ4 and Dxz4 in man and mouse (22,58).

Examination of the interval between Fcgr3 and C1orf192 indicates that like humans, the mouse genomic locus is SINE rich and contains a substantial number of LTR elements. The DNA sequence centered on the clustered tRNA genes is GC rich at 54.5% GC (Figure 8b) compared to the mouse genome average of 42.0% (62), and is enriched for LTR elements at 23.59% compared to the genome average of 9.87% (62); both are features found at the human tDNA cluster. Furthermore, pair-wise alignment of the DNA sequence clearly shows tandem arrangement of the tDNA cluster, flanked by LTR elements (Figure 8b). The mouse tandem repeat units are 7.6 kb (Figure 8c), slightly longer than the human 7.3-kb monomer (Figure 3) and are defined by XbaI. Like the human repeat unit, the mouse monomer contains numerous simple repeats as well as a partial LTR element (MalR) and a partial SINE instead of LINE element. The order and orientation of tRNA genes is also conserved between the human and mouse tandem repeat units. However, the orientation of the array is inverted relative to the flanking genes in mouse. Collectively, these data parallel the findings between the human and mouse DXZ4 tandem repeats (58).

## DISCUSSION

It is not unusual to find tRNA genes clustered in eukaryotic genomes (63,64), an arrangement that might support postmitosis reestablishment of Pol III transcriptional factories in the nucleus (65). However, outside of *Entamoeba* (66,67), arrangement of tRNA genes into homogenous tandem repeats is uncommon. Here, we describe the organization of a novel tDNA tandem repeat on human chromosome 1q23.3 that displays extensive CNV, making it the first tDNA VNTR to be described in the human genome. One implication of this observation is that individuals have the potential for variable levels of the corresponding tRNA^Leu^, tRNA^Gly^, tRNA^Glu^ and tRNA^Asp^ tRNA products, depending on their allele size, although as outlined earlier in the results, confirming this supposition might be challenging due to multiple additional copies of these genes scattered throughout the genome (55,64). Alternatively, it is possible that not all of the tDNA genes from the cluster are transcriptionally active. Chromatin signatures indicate that the tandem repeat is both heterochromatic and euchromatic, which could translate into a given number of repeat units being actively transcribed and the remainder packaged into silent chromatin. A similar phenomenon is observed at the ribosomal RNA genes, where the genes are arranged into extensive tandem arrays of which many are organized into heterochromatin that contributes to maintaining genome integrity (68).

Notably, the DNA sequence immediately proximal and distal to the tandem repeat is characterized by the presence of ERV LTR elements; a feature that is conserved in mouse. Why these sequence elements flank the tandem repeat is unclear. However, at least in yeast, LTR containing Ty1 and Ty3 retrotransposons are frequently targeted upstream of Pol III genes, corresponding to preferred insertion sites (69,70,71), and, therefore, it is conceivable that ERV LTR elements are targeted near the tDNA cluster through a similar Pol III-mediated mechanism.

The obvious purpose of tDNA is to encode tRNA molecules necessary for transporting amino acids to elongating polypeptide chains at the ribosome. However, it is becoming increasingly evident that tDNA fulfils several other functional roles in the genome besides transcription (72), including pausing the progression of replication forks (73), altering DNA access through nucleosome positioning (74), inhibition of RNA polymerase II transcription (75,76,77,78), directing tDNA subnuclear localization (79), facilitating sister chromatid cohesion (80,81,82) and contributing to the 3D organization of chromosome territories (83). Another important function attributed to tDNA is barrier activity; partitioning the genome into distinct chromatin domains by blocking heterochromatin spread. This activity, mediated through Pol III, is best characterized in *Saccharomyces cerevisiae* (78,84,85) and *Schizosaccharomyces pombe* (86,87,88,89), and can provide insulator activity by blocking *cis*-communication between promoters and enhancer elements (90,91). More recently, tDNA-associated barrier and enhancer-blocking activities have been reported in mammals (92,93). In fact, Ebersole *et al.* used part of the mouse chromosome 1qH3 tDNA cluster that we describe here to demonstrate barrier activity for tRNA genes in mice (92). Barrier activity was influenced by the orientation and copy number of the tRNA genes, and is retained for longer term if the GC-rich DNA between the tRNA genes was exchanged with AT-rich sequences. Therefore, at least *in vitro*, the tDNA tandem repeat possesses barrier activity. Should the tDNA repeat actually function as a barrier *in vivo*, the strength of the barrier activity may be influenced by the overall size of the alleles due to CNV.

The association of the epigenetic organizer protein CTCF (42) with the tDNA VNTR supports that this locus acts as a boundary element. CTCF is found throughout the human genome (60), and is enriched at the border between spatially compartmentalized chromatin interacting domains, or topological domains (94), and both the human and mouse tRNA cluster reside at a topological domain boundary (Supplemental Figure S3). Indeed, an algorithm designed to detect chromatin boundary elements in humans found both tRNA genes and CTCF as common predictive features of boundaries (95). Evidence that support an important role for CTCF at the tDNA VNTR comes from comparing the sequences of the human 7.3-kb and mouse 7.6-kb tandem repeat units. Despite their similar size, the only DNA sequence that is conserved (with the notable exception of the tRNA gene sequences) is a 24 bp sequence that corresponds to the CTCF/Ctcf binding motif, indicating that this sequence is under selective pressure to be retained. CTCF mediates long-range interactions and is central to compartmentalizing the genome and organizing chromatin domains (96). CTCF also associates with the X-linked macrosatellite DXZ4 (22,97), and like the mouse and human tDNA VNTR homologues, DNA sequence conservation between human DXZ4 and mouse Dxz4 is restricted to a short DNA sequence corresponding to the CTCF/Ctcf binding motif (58). At least in humans, DXZ4 makes frequent long-range chromosomal interactions with other CTCF-bound tandem repeats on the inactive X chromosome and is a candidate chromosomal folding element that may account for the alternate 3D organization of the inactive X chromosome territory (24). This activity appears to be dependent upon CTCF, as depletion of protein levels significantly reduces interactions (23). Therefore, it is conceivable that the chromosome 1q23.3 tDNA VNTR is also involved in mediating long-range interactions and higher order chromosome organization, and is not simply a tRNA gene cluster.

Several other interesting parallels can be drawn between the tDNA VNTR and DXZ4. These include (i) both are GC-rich extensive VNTRs (6,10,12), (ii) both are characterized by euchromatin and heterochromatin markers and (iii) both reside in a region of conserved gene order (58). Why retain a GC-rich homogenous tandem repeat, when 94%, corresponding to almost 7-kb of each repeat unit, is not conserved between man and mouse? This suggests that the overall size of the array as well as tandem arrangement, are as important for function as the presence of the tRNA genes and the CTCF/Ctcf binding site. Furthermore, both the mouse and human tandem repeat units are enriched for CpG (588 CpG per human repeat unit and 356 in each mouse repeat unit) making them extensive CGIs. Many CGIs correspond to regulatory elements in the genome (98). Evidence supporting a regulatory role for the tDNA VNTR comes from the fact that in hESCs the locus bears the hallmarks of a ‘bivalent’ domain: marked by the simultaneous presence of both H3K4me3 and H3K27me3 chromatin modifications (52). Bivalent domains are thought to perform an important role during development. Whether this tDNA VNTR functions beyond simply providing tRNA product remains an open question and warrants further investigation.

## SUPPLEMENTARY DATA

Supplementary Data are available at NAR Online.

## FUNDING

National Institutes of Health (NIH) [GM073120 to B.P.C.]. Funding for open access charge: NIH [GM073120].

*Conflict of interest statement*. None declared.

## Supplementary Material

SUPPLEMENTARY DATA
