# Supplementary material for: A novel tRNA variable number tandem repeat at human chromosome 1q23.3 is implicated as a boundary element based on conservation of a CTCF motif in mouse
Source: Nucleic Acids Res. 2014 Apr 21;42(10):6421–35. doi: 10.1093/nar/gku280 (PMC4041453; doi:10.1093/nar/gku280)
Supplement: SUPPLEMENTARY DATA [file supp_42_10_6421__index.html]

A novel tRNA variable number tandem repeat at human chromosome 1q23.3 is implicated as a boundary element based on conservation of a CTCF motif in mouse — SUPPLEMENTARY DATA 

# A novel tRNA variable number tandem repeat at human chromosome 1q23.3 is implicated as a boundary element based on conservation of a CTCF motif in mouse

## SUPPLEMENTARY DATA

**Files in this Data Supplement:**

- SUPPLEMENTARY DATA
