# Supplementary material for: A novel tRNA variable number tandem repeat at human chromosome 1q23.3 is implicated as a boundary element based on conservation of a CTCF motif in mouse
Source: Nucleic Acids Res. 2014 Apr 21;42(10):6421–35. doi: 10.1093/nar/gku280 (PMC4041453; doi:10.1093/nar/gku280)
Supplement: SUPPLEMENTARY DATA [file supp_gku280_nar-00435-h-2014-File010.pdf]

Supplementary Material

Supplementary Figure 1

Genomic Southern blot showing the hybridization results for the Chr1 Dig-labeled probe. The blot consists of human genomic DNA digested with *Eco*RI before separation on a 0.8% agarose gel. DNA size based on the marker lane is shown to the right. The unique 7.3kb band corresponding to a single repeat monomer is indicated to the left. The blot was hybridized with the DIG-labeled probe for 3 hours at 60°C in ExpressHyb (Clontech Laboratories Inc., Mountain View, CA, USA), before washing at 60°C for 8 minutes twice with wash one (2x SSC, 0.1% SDS) and twice with wash two (0.2x SSC, 0.1% SDS). The signal was detected using the DIG High Prime DNA Labeling and Detection Starter Kit II according to the manufacturers instructions (Roche Applied Science, Indianapolis, IN, USA).

Chr1 Probe Specificity Check

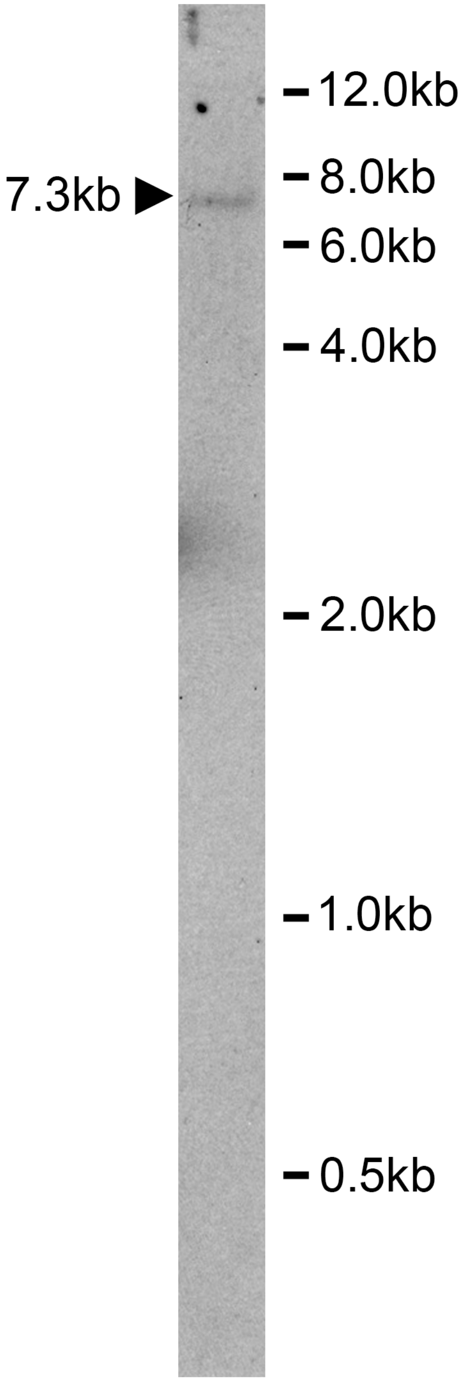

Supplementary Figure 2

Comparison of the consensus CTCF binding motif obtained adapted from JASPAR (100), to a probability map of the conserved human and mouse sequence from the tDNA repeat that underlies the human CTCF ChIP-Seq peaks. The probability plot was generated using WebLogo (101), and used the reverse complement of the human and mouse sequences.

Jaspar Core

Human
& Mouse
tDNA

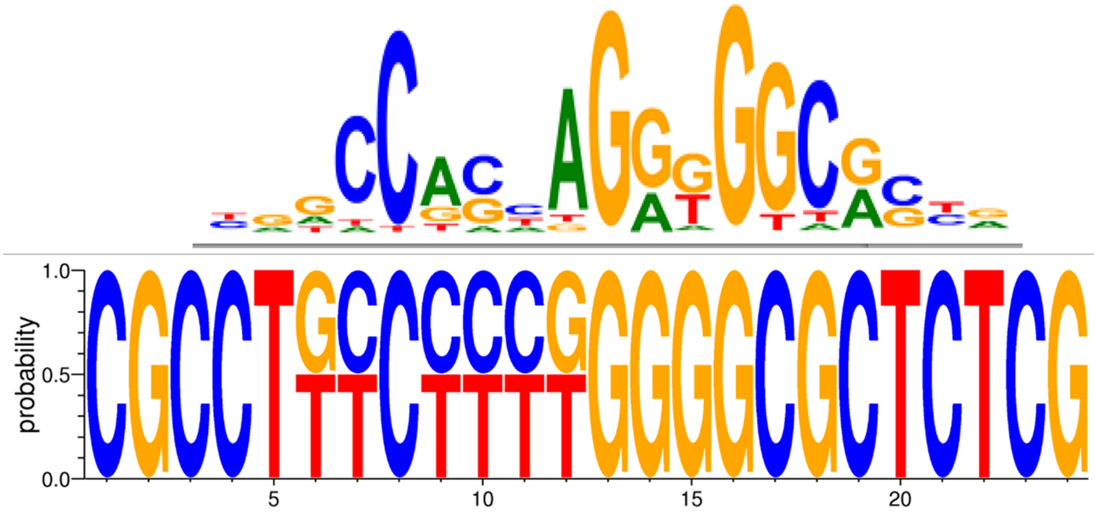

Supplementary Figure 3

Chromatin features of the mouse tDNA repeat. The black arrow represents a single monomer at the top of the image, under which the open arrows indicate the location of the tRNA genes. Below this are ChIP-Seq and input control profiles for various mouse cell and tissue types adapted from the UCSC Genome Browser (http://genome.ucsc.edu/index.html).

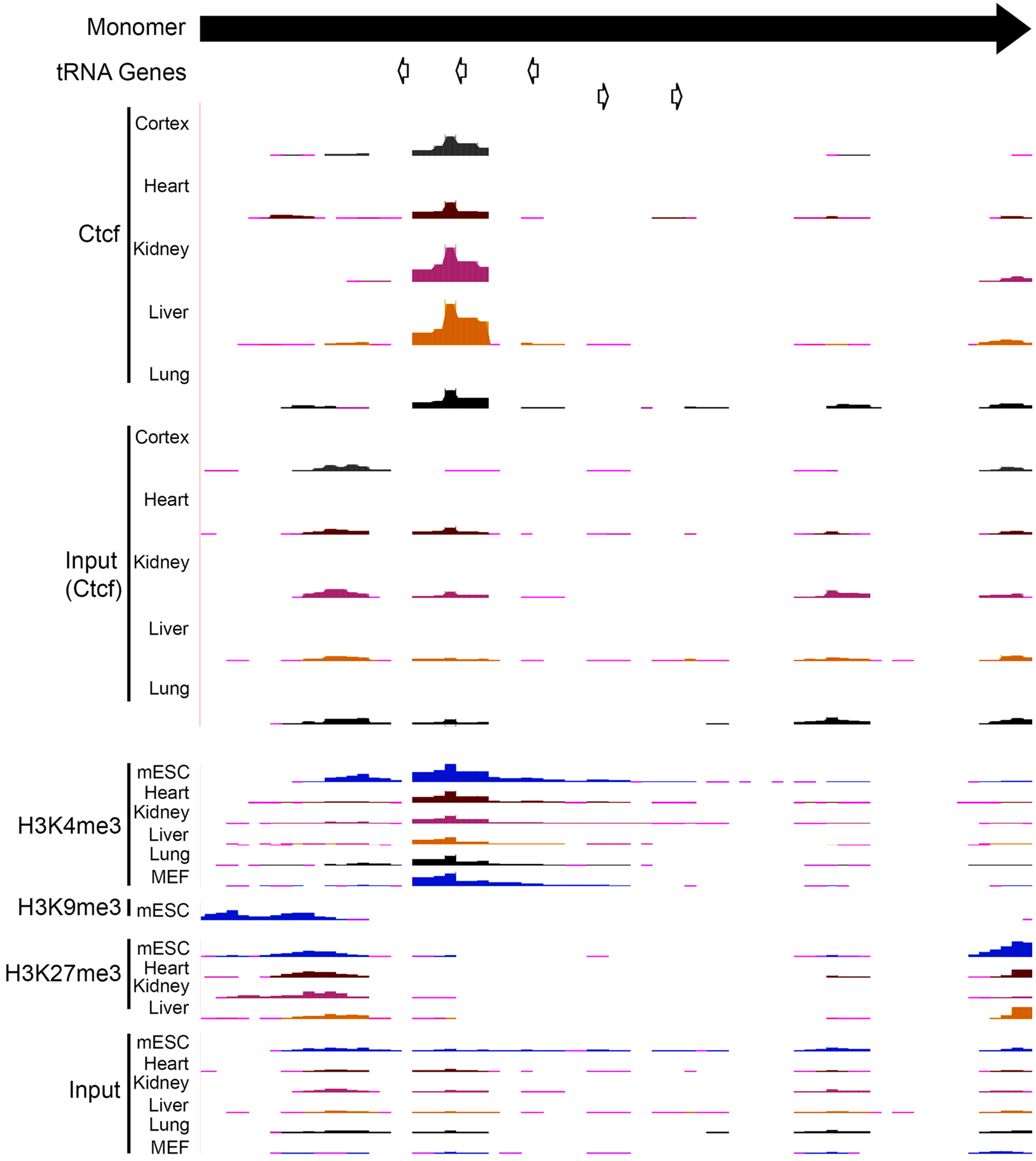

Monomer
tRNA Genes
Ctcf
Cortex
Heart
Kidney
Liver
Lung
Input (Ctcf)
Cortex
Heart
Kidney
Liver
Lung
H3K4me3
mESC
Heart
Kidney
Liver
Lung
MEF
H3K9me3
mESC
H3K27me3
mESC
Heart
Kidney
Liver
Input
mESC
Heart
Kidney
Liver
Lung
MEF

Supplementary Figure 4

Schematic map showing the relationship of the Human and Mouse tDNA tandem repeats (right-facing black arrows) relative to topological domains, represented by the green and red genomic compartments (93). The image was adapted from the HiC Project at the Ren Lab (http://yuelab.org/hi-c/). White arrows indicate the location of transcripts from the interval, oriented in the direction of transcription and covering the approximate genomic span of the corresponding gene. Human data is shown for human embryonic stem cells (hESC) and lung fibroblast IMR90, and corresponds to 159,520,867-159,880,006 of human chromosome 1 (build hg18). Mouse data is shown for mouse embryonic stem cells (mESC) and mouse cortex, and corresponds to 172,880,561-173,119,754 of mouse chromosome 1 (build mm9).

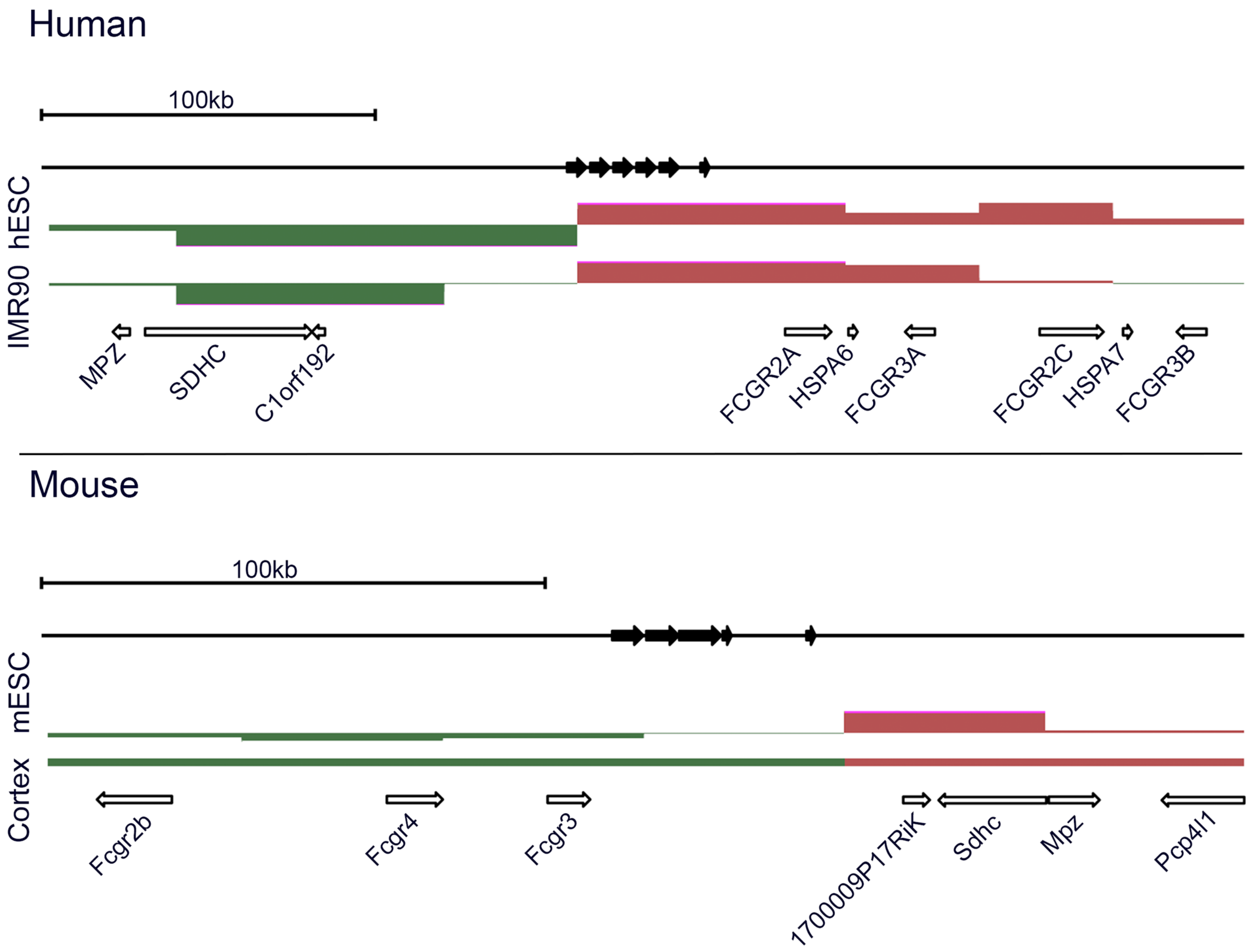
Human
100kb
IMR90 hESC
MPZ
SDHC
C1orf192
FCGR2A
HSPA6
FCGR3A
FCGR2C
HSPA7
FCGR3B
Mouse
100kb
Cortex mESC
Fcgr2b
Fcgr4
Fcgr3
1700009P17RiK
Sdhc
Mpz
Pcp4l1
